# Negative Effect of *Gst‐35* on the Health Span of *Caenorhabditis elegans* Through Lysosomal Dysfunction via the *Pmk‐1* and *Skr* Genes

**DOI:** 10.1111/acel.70016

**Published:** 2025-02-13

**Authors:** Yehui Gao, Xinyun Zhang, Congmin Wei, Hongru Lin, Mengchen Wu, Botian Ma, Jinyun Jiang, Shan Li, Hongbing Wang

**Affiliations:** ^1^ School of Life Sciences and Technology, Shanghai East Hospital, Institute for Regenerative Medicine Tongji University Shanghai China; ^2^ Primary Education College Shanghai Normal University Tianhua College Shanghai China; ^3^ Tongji Alpha Natural Medicine Research Institute Tongji University Shanghai China

**Keywords:** *Caenorhabditis elegans*, *gst‐35*, health span, lysosomal dysfunction

## Abstract

As global life expectancy increases, the focus has shifted from merely extending lifespan to promoting healthy aging. GSTA1, GSTA2, and GSTA3 (GSTA1‐3), members of the alpha class of glutathione S‐transferases, are involved in diverse biological processes, including metabolism and immune regulation, highlighting their potential influence on human health span and lifespan. In this study, we employed *Caenorhabditis elegans* as a model organism to investigate the role of *gst‐35*, an ortholog of mammalian GSTA1‐3, in healthy aging. Our results demonstrated that *gst‐35* overexpression has detrimental effects on multiple physiological functions in nematodes. Specifically, *gst‐35* overexpression significantly reduced lifespan, impaired development and growth, and substantially diminished reproductive capacity, physical fitness, and stress resistance. In contrast, *gst‐35* knockout partially enhanced physical fitness and stress resistance. Comprehensive RNA‐sequencing transcriptome analysis revealed that *gst‐35* overexpression disrupted metabolic homeostasis and induced lysosomal dysfunction. These effects were mediated through the activation of the *pmk‐1* signaling pathway and suppression of *skr* genes, which collectively impaired healthy aging processes. These findings illuminate the complex role of *gst‐35* in aging and provide valuable insights into the molecular mechanisms underlying healthy aging, offering potential targets for interventions aimed at promoting health span.

## Graphical abstract

The overexpression of *gst‐35*, the mammalian orthologous genes GSTA1, GSTA2, and GSTA3, induces lysosomal dysfunction through the genes *pmk‐1* and *skr* in *C. elegans* . This overexpression not only promotes an in vivo inflammatory response and accumulation of reactive oxygen species but also significantly shortens the nematode lifespan; inhibits its development and growth; reduces reproduction, physical fitness, and stress resistance; and disrupts metabolic homeostasis, ultimately leading to impaired healthy lifespan.

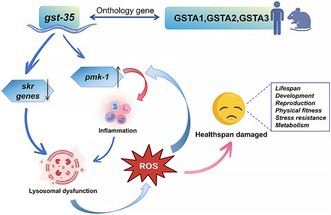

**Abbreviations**
AD: Alzheimer's disease
C. elegans: Caenorhabditis elegans
FUDR: 5‐fluoro‐2′‐deoxyuridine
GO: Gene Ontology
GSH/GSSG: reduced and oxidized glutathione ratio
GST: glutathione S‐transferase
*gst‐35*oe: overexpression of *gst‐35*
GSTA1‐3: GSTA1, GSTA2, and GSTA3
KEGG: Kyoto Encyclopaedia of Genes and Genome
KO: knockout
LB: Luria Bertani
MAPK: mitogen–activated protein kinase
NGM: nematode growth medium
OE: overexpression
PA14: *Pseudomonas aeruginosa*
PBS: phosphate‐buffered saline
PCR: polymerase chain reaction
PD: Parkinson's disease
PGD2: prostaglandin D2
qPCR: quantitative PCR
RNAi: RNA interference
RNA‐seq: RNA sequencing
ROS: reactive oxygen species
SKP1: S‐phase kinase associated protein 1
*skr‐10*oe: overexpression of *skr‐10*
WT: wild type

## 1 Introduction

Aging, a fundamental biological process, is characterized by a progressive decline in cellular and physiological integrity, ultimately leading to reduced overall well‐being (Leidal et al. 2018). Although global human lifespans have increased, this extension has not always been accompanied by a corresponding improvement in health span, resulting in a rise in age‐related diseases and morbidities (Campisi et al. 2019). The importance of prioritizing health span alongside lifespan was emphasized during the Geoscience Network assembly, which highlighted that extending lifespan alone does not necessarily delay the aging process (Rollins et al. 2017). Therefore, developing interventions that promote healthy aging by enhancing both health span and lifespan is essential.

The goal of promoting health span is to improve life quality by alleviating the effects of age‐related disorders and mitigating health deterioration. This involves preserving youthful physiological functions, including maintaining activities such as exercise and feeding behavior, as well as reducing the accumulation of lipofuscin within cells (Gerstbrein et al. 2005; Huang et al. 2004; Lee et al. 2006). Additionally, research has revealed that health span can be extended through evolutionarily conserved genes and mechanisms (Bansal et al. 2015). However, the exact mechanisms by which these genes influence lifespan remain poorly understood. Notably, studies have shown that certain long‐lived mutants of *Caenorhabditis elegans* ( *C. elegans* ), unlike their wild‐type (WT) counterparts, spend a larger proportion of their extended lifespan in a frail state (Bansal et al. 2015). This indicates that although these nematodes may achieve increased longevity, their overall health span is not necessarily improved. Therefore, in the pursuit of extending lifespan, it is crucial to prioritize concurrent improvements in life quality.

GSTA1, GSTA2, and GSTA3 (GSTA1‐3) are members of the alpha class of glutathione S‐transferase (GST) (Strange et al. 2001). Their expression is age dependent, with a significant upregulation observed in age‐related liver dysfunction (He et al. 2020). Previous research has shown that the expression levels of GSTA1‐3 increase with age in mice (Patel et al. 1998). Additionally, growing evidence suggests that GSTA1‐3 contributes to a heightened risk of conditions such as asthma and ovarian cancer (Khrunin et al. 2010; Kishore et al. 2020; Polimanti et al. 2010). Specifically, GSTA3 has been shown to promote the production of prostaglandin D2 (PGD2) (Niu et al. 2021), a key mediator in the development of asthma and inflammatory responses (Mohri et al. 2003). These findings highlight the diverse biological roles of GSTA1‐3, including metabolism and immune regulation, while underscoring its potentially detrimental effects on human health span and lifespan. Nevertheless, the precise mechanisms by which GSTA1‐3 influences health span remain rarely elucidated.

*C. elegans* is a well‐established model organism for aging research due to its short lifespan, genetic tractability, and the conservation of key genes and signaling pathways related to aging and immunity. According to WormBase, a comprehensive database for the *C. elegans* genome (www.wormbase.org), *gst‐35*, a homolog of mammalian GSTA1‐3, is involved in glutathione metabolism, cellular detoxification, and oxidative stress response. It plays a critical role in maintaining intracellular homeostasis (Kishore et al. 2020; van de Wetering et al. 2021). Despite its importance, there is a lack of comprehensive studies examining the impacts and underlying mechanisms of *gst‐35* on health span and lifespan.

To evaluate the impact of *gst‐35* on the health span of *C. elegans* , we assessed several health span markers, including growth and developmental stages, reproductive capacity, muscular function, stress resistance, and the accumulation of lipofuscin. Our findings indicated that reducing *gst‐35* expression improves the health status of nematodes without affecting their lifespan. In contrast, overexpression (OE) of *gst‐35* (*gst‐35*oe) significantly decreased lifespan and was associated with deteriorated health indicators. Furthermore, this study revealed that *gst‐35* expression increases with age. *Gst‐35*oe was found to impair lysosomal function by activating the *pmk‐1* signaling pathway and reducing the expression of *skr* genes, leading to inflammation, metabolic dysregulation, and a decline in the health span. These findings provided new insights into the role of *gst‐35* in modulating the lifespan and health of *C. elegans* , offering a deeper understanding of the potential functions of GSTA1‐3 in similar processes.

## 2 Results

### 2.1 *Gst‐35*oe Shortened the Lifespan of *C. elegans*

**FIGURE 1:**
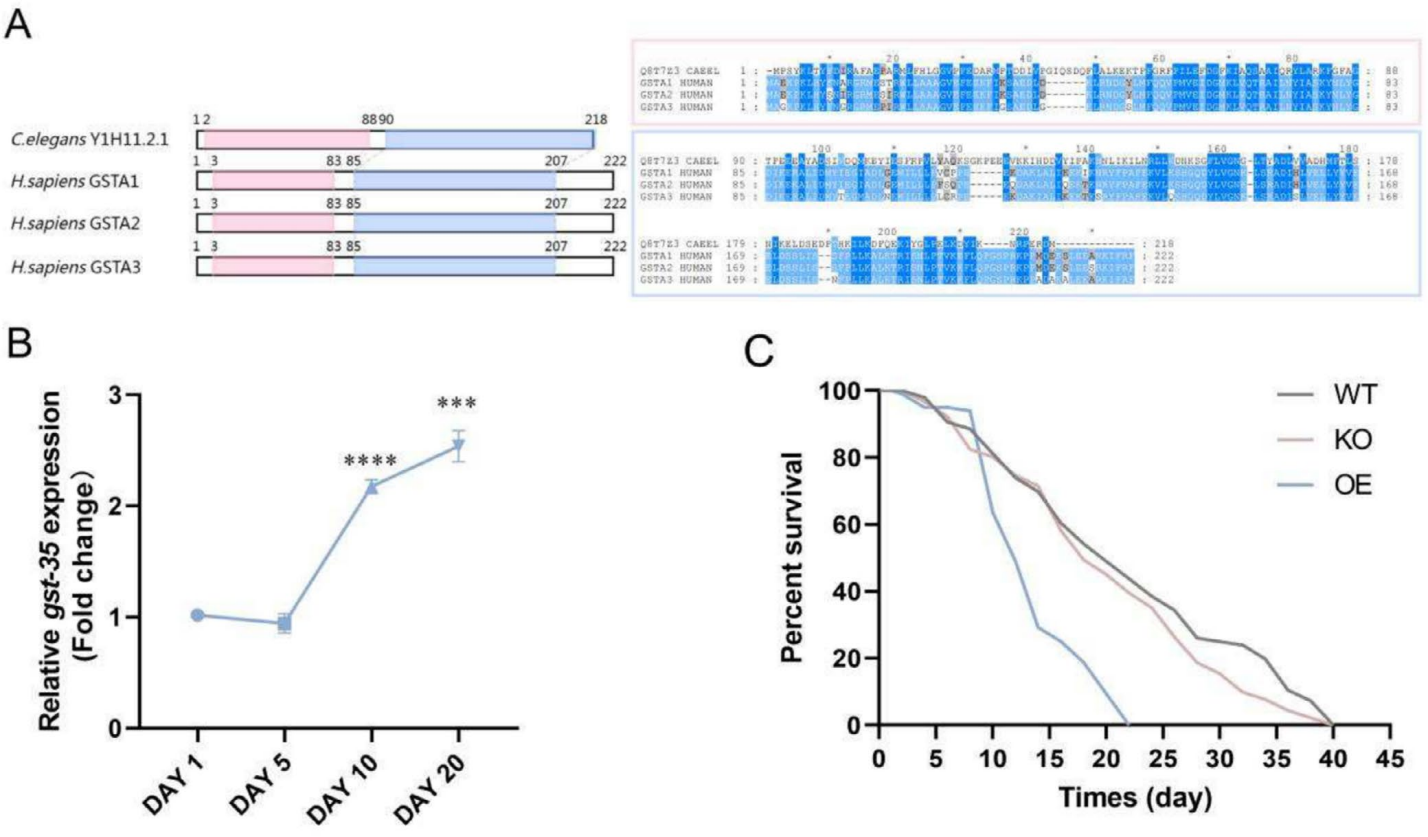
*Gst‐35*oe reduces the lifespan of *C. elegans* . (A) Left, schematic representation of the two evolutionarily conserved regions in GSTA1‐3 human orthologs and in *C. elegans* Y1H11.2.1. The N‐terminal and C‐terminal regions are shaded in pink and blue, respectively. Right, multiple sequence alignments corresponding to these two conserved regions represented within colored boxes in pink and blue, respectively. The amino acid coloring scheme indicates the average BLOSUM62 score (correlated to amino acid conservation) in each alignment column: blue (greater than 3.5), light blue (between 3.5 and 1.5), and gray (between 1.5 and 0.5). Sequences are labeled according to their UniProt identifier. GSTA1_HUMAN, GSTA2_HUMAN, GSTA3_HUMAN, *Homo sapiens* ; Q8T7Z3_CAEEL, *C. elegans* . (B) The expression levels of *gst‐35* mRNA were assessed at multiple time points (Days 1, 5, 10, and 20) in wild‐type (WT) nematodes. (C) The survival curves of WT, knockout (KO), and overexpression (OE) nematodes are shown. Data were analyzed using the Student's *t*‐test with Prism 8.0. Values represent mean ± SEM of three independent experiments. ***p < 0.001; ****p < 0.0001.

We first analyzed the conservation of *gst‐35* and GSTA1‐3 across species using multiple sequence alignment. This analysis identified two evolutionarily conserved domains within the protein encoded by the *Y1H11.2.1* gene, showing strong conservation between humans and *C. elegans* (Figure 1A). Next, we observed a significant age‐dependent increase in *gst‐35* mRNA expression levels in WT nematodes (Figure 1B). To investigate the role of *gst‐35* in *C. elegans* longevity, we constructed knockout (KO) strain PHX6971 and OE strain PHX7528. Lifespan analysis revealed a 36.12% reduction in the lifespan of the OE strain compared to WT nematodes (Figure 1C and Table 1). In contrast, the KO strain exhibited no significant change in lifespan. These findings indicated that *gst‐35* plays a critical role in modulating the lifespan of *C. elegans* and that *gst‐35*oe significantly shortens their lifespan.

**TABLE 1:** Lifespan of wild‐type (WT), knockout (KO), and overexpression (OE) nematodes at 20°C.

Strain	Number of nematodes	Mean lifespan (day)	Change in lifespan (%)
WT	282	21.02 ± 0.47	
KO	275	21.71 ± 0.80^ns^	3.28
OE	308	13.42 ± 0.14***	−36.12

*Note:* The statistical difference between the curves was analyzed using the log‐rank test.

*** *p* < 0.001 vs. WT. ns indicates no statistical significance vs. WT.

### 2.2 *Gst‐35*oe Impaired the Development, Growth, and Reproductive Ability of *C. elegans*

**FIGURE 2:**
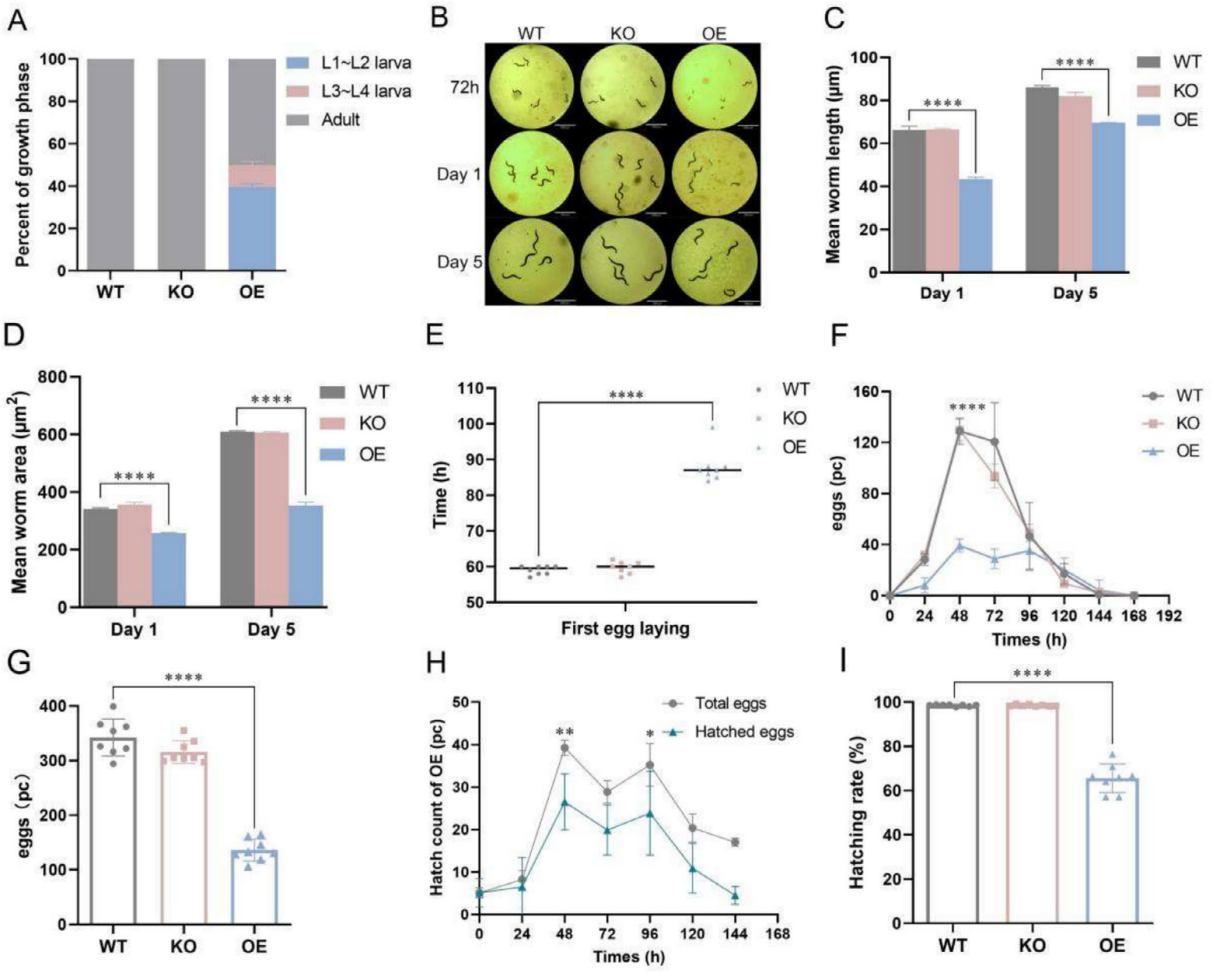
*Gst‐35*oe inhibits development, growth, and reproductive capacity in *C. elegans*. (A) Bar chart illustrating the development phase in WT, KO, and OE nematodes on Day 1. (B–D) The body length and body area of WT, KO, and OE nematodes on Days 1 and 5. (E) The first egg laying time of WT, KO, and OE nematodes. (F) The daily egg production of WT, KO, and OE nematodes. (G) The total number of eggs laid by WT, KO, and OE nematodes. (H) The total and daily hatched eggs of the OE nematodes. (I) The hatched rate of eggs of WT, KO, and OE nematodes. Data were represented as mean ± SEM. *p < 0.05; **p < 0.01; ****p < 0.0001 vs. WT.

During the lifespan study, we observed asynchronous development in the OE groups compared to the WT groups. Specifically, 51.16% of OE nematodes failed to reach the adult stage (Figure 2A), indicating that *gst‐35*oe negatively impacted their developmental progression. In contrast, KO nematodes showed no adverse effects on development. To assess the impact of *gst‐35* on nematode growth, we used the WormLab system to quantify body length and area at Days 1 and 5. *Gst‐35*oe significantly inhibited both body length and area, whereas KO nematodes showed no significant changes in these parameters (Figure 2B–D). Given the complex relationship between growth, development, and reproductive capacity (Zhao et al. 2023), we further examined the effect of *gst‐35* on the reproductive potential of nematodes. *Gst‐35*oe significantly impaired the reproductive capacity, as indicated by a marked reduction in the number of eggs laid and a delayed onset of egg production (Figure 2E and Table S2). The OE strain showed a 29.00‐h delay in the initiation of egg laying, correlating with the observed developmental asynchrony in these mutants (Figure 2A). Furthermore, egg production in the OE strain was significantly reduced compared to the WT and KO groups, both daily and over the entire reproductive period (Figure 2F,G). On average, the OE strain produced only 136.30 eggs (Table S2). Additionally, the hatching rate of eggs in the OE strain progressively declined over time, with only 65.60% of eggs successfully hatching (Figure 2H,I and Table S2). These findings suggested that *gst‐35*oe adversely affects the growth, development, and reproductive capacity of *C. elegans* .

### 2.3 *Gst‐35*oe Damaged Physical Fitness, Increased Oxidative Stress, and Induced Inflammation in *C. elegans*

**FIGURE 3:**
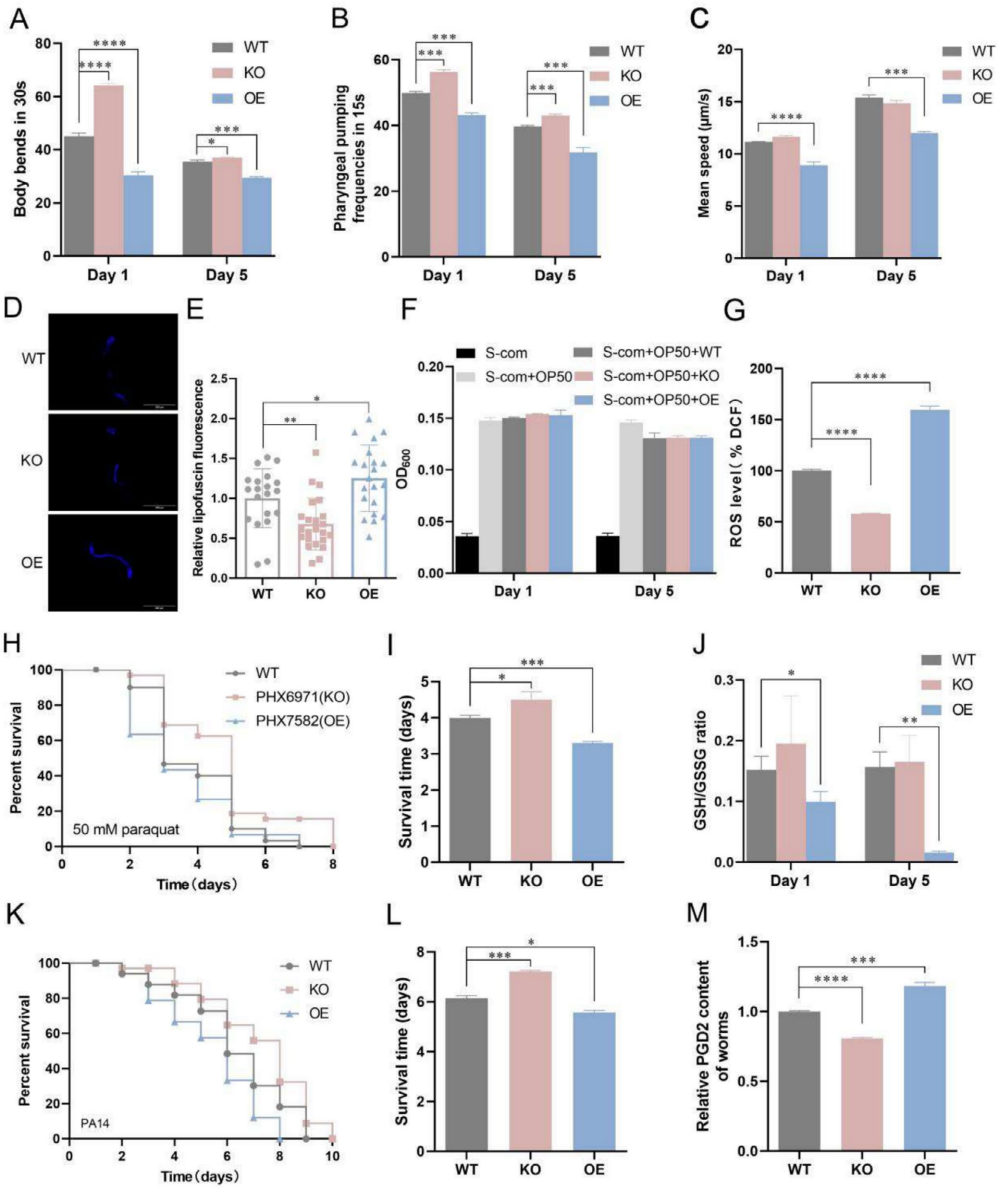
*Gst‐35*oe hampers the physiological function of nematodes. (A–C) Effect of *gst‐35* on body bending, pharyngeal pumping rate, and moving speed on Days 1 and 5 of adulthood. (D, E) Effect of *gst‐35* on lipofuscin accumulation in nematodes on Day 5. (F) Effect of *gst‐35* on the feeding amount of nematodes. (G) Intracellular ROS levels in WT, KO, and OE nematodes. (H, I) The survival curves and bar charts of WT, KO, and OE nematodes exposed to 50 mM paraquat on Day 1. (J) The GSH/GSSG ratio in WT, KO, and OE nematodes on Days 1 and 5. (K, L) The survival curves and bar charts of WT, KO, and OE nematodes exposed to PA14 on Day 1. (M) The content of prostaglandin 2 (PGD2) in WT, KO, and OE nematodes on Day 5. Data were analyzed with the Student's *t*‐test using Prism 8.0. Values represent mean ± SEM of three independent experiments. *p < 0.05; **p < 0.01; ***p < 0.001; ****p < 0.0001.

*Gst‐35*oe shortened the lifespan and negatively impacted the fitness of *C. elegans* . On Days 1 and 5, the body bends, pharyngeal pumping rate, and mean speed were significantly decreased in the OE nematodes compared to the WT nematodes. In contrast, KO nematodes exhibited a significant increase in these fitness parameters (Figure 3A–C). Additionally, *gst‐35*oe led to a significant increase in lipofuscin content, a well‐known aging biomarker (Gray and Woulfe 2005), whereas *gst‐35* KO had the opposite effect (Figure 3D,E), suggesting that *gst‐35* might accelerate the aging process in nematodes. However, neither OE nor KO of *gst‐35* had any effect on the food intake capacity of the nematodes (Figure 3F).

The GST family facilitates the formation of glutathione‐S conjugates by binding with GSH, leading to the consumption of total intracellular GSH (Dringen 2000). GSH functions as a critical intracellular antioxidant, maintaining redox homeostasis by scavenging reactive oxygen species (ROS) (Hellou et al. 2012). The reduced and oxidized glutathione (GSH/GSSG) ratio serves as an important indicator of the redox environment and cellular health (Ferguson and Bridge 2019). In our study, we observed significantly increased ROS levels in the OE nematodes and decreased ROS levels in the KO nematodes compared to the WT group (Figure 3G). Compared to the WT group, the survival rate of OE nematodes treated with 50 mM paraquat decreased, whereas the survival rate of KO nematodes increased (Figure 3H,I). Additionally, the GSH/GSSG ratio was markedly reduced in the OE nematodes on both Days 1 and 5 (Figure 3J).

Elevated ROS levels can cause oxidative damage and apoptosis, potentially triggering inflammation during immune responses or in pathological conditions (Yang and Lian 2020). The homologous gene GSTA3 has been implicated in asthma development through the promotion of PGD2 production, a pro‐inflammatory mediator (Niu et al. 2021). To explore the anti‐inflammatory potential of *gst‐35* and assess its inflammatory status, we infected the nematodes with *Pseudomonas aeruginosa* (PA14) and measured the PGD2 levels. The OE nematodes exhibited increased susceptibility to PA14 infection, indicating weakened immune function (Figure 3K,L). Furthermore, PGD2 levels were significantly higher in the OE nematodes, whereas the KO nematodes showed reduced PGD2 levels, suggesting heightened inflammatory responses in the OE nematodes (Figure 3M). These findings suggested that *gst‐35*oe induces ROS accumulation, which stimulates the production of the inflammatory mediator PGD2, potentially triggering an inflammatory state that negatively impacts the lifespan and health of nematodes.

### 2.4 Negative Effects of *Gst‐35*oe Are Closely Related to Lysosome Dysfunction

**FIGURE 4:**
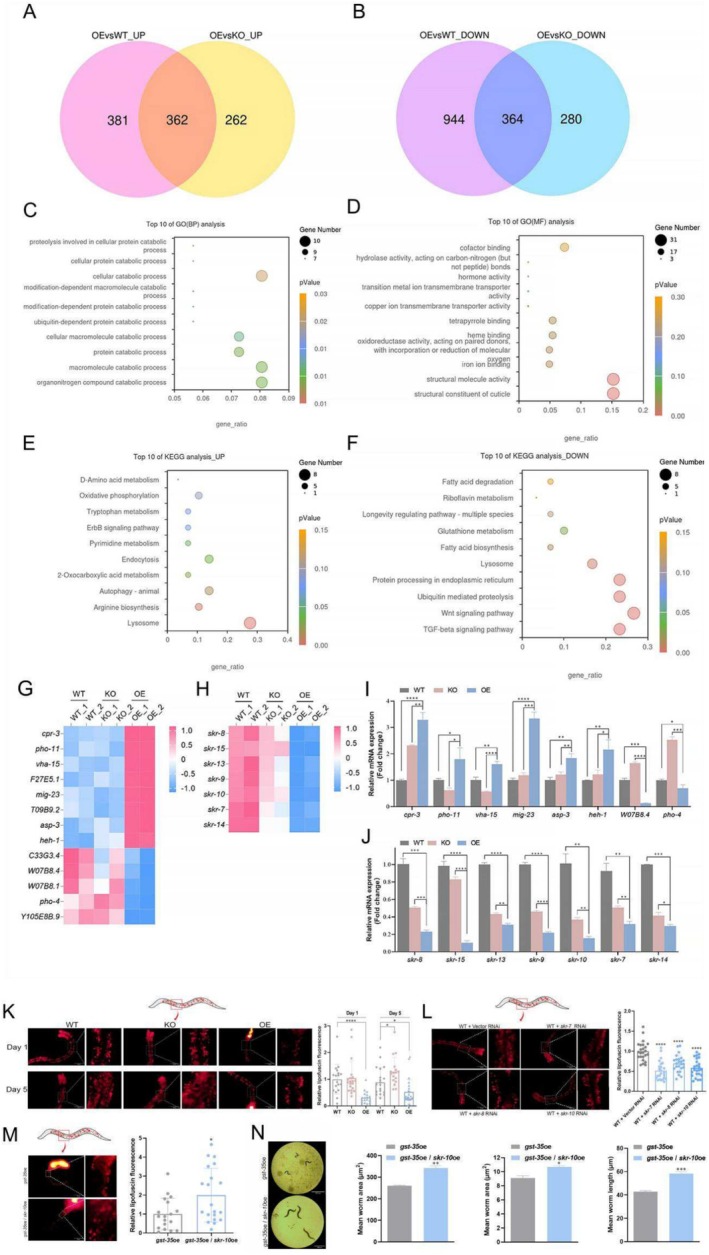
*Gst‐35*oe disrupts lysosomal activity. (A, B) A Venn diagram to compare the gene expression profiles of OE versus WT nematodes and OE versus KO nematodes (*p* < 0.05). RNA‐seq was performed on nematodes on Day 5 to determine the gene expression patterns (*N* = 2). (C, D) A Gene Ontology (GO) enrichment analysis chart was generated to visualize the top 10 terms associated with biological processes and molecular functions. The color represents enrichment significance, and the bubble size represents gene count. (E, F) A Kyoto Encyclopaedia of Genes and Genome (KEGG) enrichment analysis chart was generated to illustrate the KEGG terms enriched by the co‐upregulated and co‐downregulated differentially expressed genes in OE compared with WT and KO nematodes, respectively. The color represents enrichment significance, and the bubble size represents gene count. (G, H) Differential gene heat maps of WT, KO, and OE nematodes were presented to visualize the patterns of gene expression changes in lysosomes and the orthologous genes of SKP1 in *C. elegans* . (I, J) Validation of differentially expressed genes screened with qPCR. (K) Representative fluorescence images of WT, KO, and OE nematodes stained with LysoTracker Red on Days 1 and 5, with its quantitative determination of acidic fluorescence intensity in the intestine. (L) Representative fluorescence images of WT nematodes, which were treated with vector L4440, *skr‐7, skr‐8* or *skr‐10* bacteria and stained with LysoTracker Red on Day 1, with its quantitative determination of acidic fluorescence intensity in the intestine. (M) Representative fluorescence images of *gst‐35*oe (OE nematodes) and *gst‐35*oe/*skr‐1*0oe nematodes stained with LysoTracker Red on Day 1, with its quantitative determination of acidic fluorescence intensity in the intestine. (N) The body length, body area, and moving speed of *gst‐35*oe (OE nematodes) and *gst‐35*oe/*skr‐10*oe nematodes on Day 1. Data were analyzed with the Student's *t*‐test using Prism 8.0. Values represent mean ± SEM of three independent experiments. *p < 0.05; **p < 0.01; ***p < 0.001; ****p < 0.0001.

To investigate the molecular mechanism by which *gst‐35* influences the health span of nematodes, we performed an RNA sequencing (RNA‐seq) transcriptome analysis to identify differentially expressed genes among the OE, KO, and WT groups. This analysis identified 362 upregulated and 364 downregulated genes in the OE nematodes (Figure 4A,B). Gene Ontology (GO) enrichment analysis revealed that *gst‐35* was extensively involved in biological processes, such as organonitrogen compound, macromolecule, and protein catabolic processes (Figure 4C). Enriched GO terms also included the structural constituent of cuticle and structural molecule activity (Figure 4D), critical for regulating growth and development (Zeng et al. 2023). Additionally, terms such as iron ion binding, oxidoreductase activity, and heme binding (Figure 4D) were linked to metabolism, oxidative stress, growth and development, and immune system (Emerit et al. 2001; Hou et al. 2006; Kawabata 2022; Zygiel and Nolan 2019). Collectively, these findings suggested that *gst‐35* played a multifaceted role in regulating growth, development, and reproduction in *C. elegans* by influencing metabolism, structural composition, and redox balance.

Kyoto Encyclopaedia of Genes and Genome (KEGG) pathway enrichment analysis revealed that the differentially expressed genes were significantly associated with lysosomal function (Figure 4E,F). Notable changes were observed in lysosomal gene expression (Figure 4G) and were validated through quantitative polymerase chain reaction (qPCR) (Figure 4I). Lysosomes, as essential organelles for maintaining cellular homeostasis, metabolic regulation, and immune function, are known to become dysfunctional with aging (Kaushik et al. 2021), contributing to various diseases, including autoimmune and metabolic disorders (Deretic 2021). To assess the impact of *gst‐35* on lysosomal function, we used LysoTracker Red dye staining to monitor lysosomal activity. Results showed a significant reduction in lysosomal activity in OE nematodes compared to WT and KO groups (Figure 4K). These findings indicated that *gst‐35* might negatively affect nematode health by disrupting lysosomal function.

In addition to the lysosomal pathways, enriched downregulated genes were linked to the TGF‐β, Wnt, and ubiquitin‐mediated proteolysis signaling pathways (Figure 4F). Most of these genes were *skr* genes, orthologs of the mammalian S‐phase kinase–associated protein 1 (SKP1) gene (Figure 4H and Table S3). qPCR analysis confirmed reduced *skr* mRNA levels in OE nematodes (Figure 4J). SKP1 is a pivotal gene in these pathways, and its phosphorylation promotes autophagy, a key process for protein degradation and secretion in lysosomes (Li, Krause, et al. 2023; Liao et al. 2004; Sakamoto et al. 2001). Consistent with previous findings (Nayak et al. 2002), silenced *skr‐7, skr‐8* and *skr‐10* in *C. elegans* delayed growth and development (Figure S1) and reduced lysosomal activity (Figure 4L). Notably, OE of *skr‐10* (*skr‐10*oe) could mitigate the physiological impairments and lysosomal dysfunction caused by *gst‐35*oe (Figure 4M), improving physiological metrics (Figure 4N). These results demonstrated that *gst‐35*oe downregulates *skr* genes, impairing lysosomal function and adversely affecting metabolism and overall nematode health.

### 2.5 *Gst‐35*oe Led to a Reduction in Lysosome Function in *C. elegans* Through the Activation of the *Pmk‐1* Pathway

**FIGURE 5:**
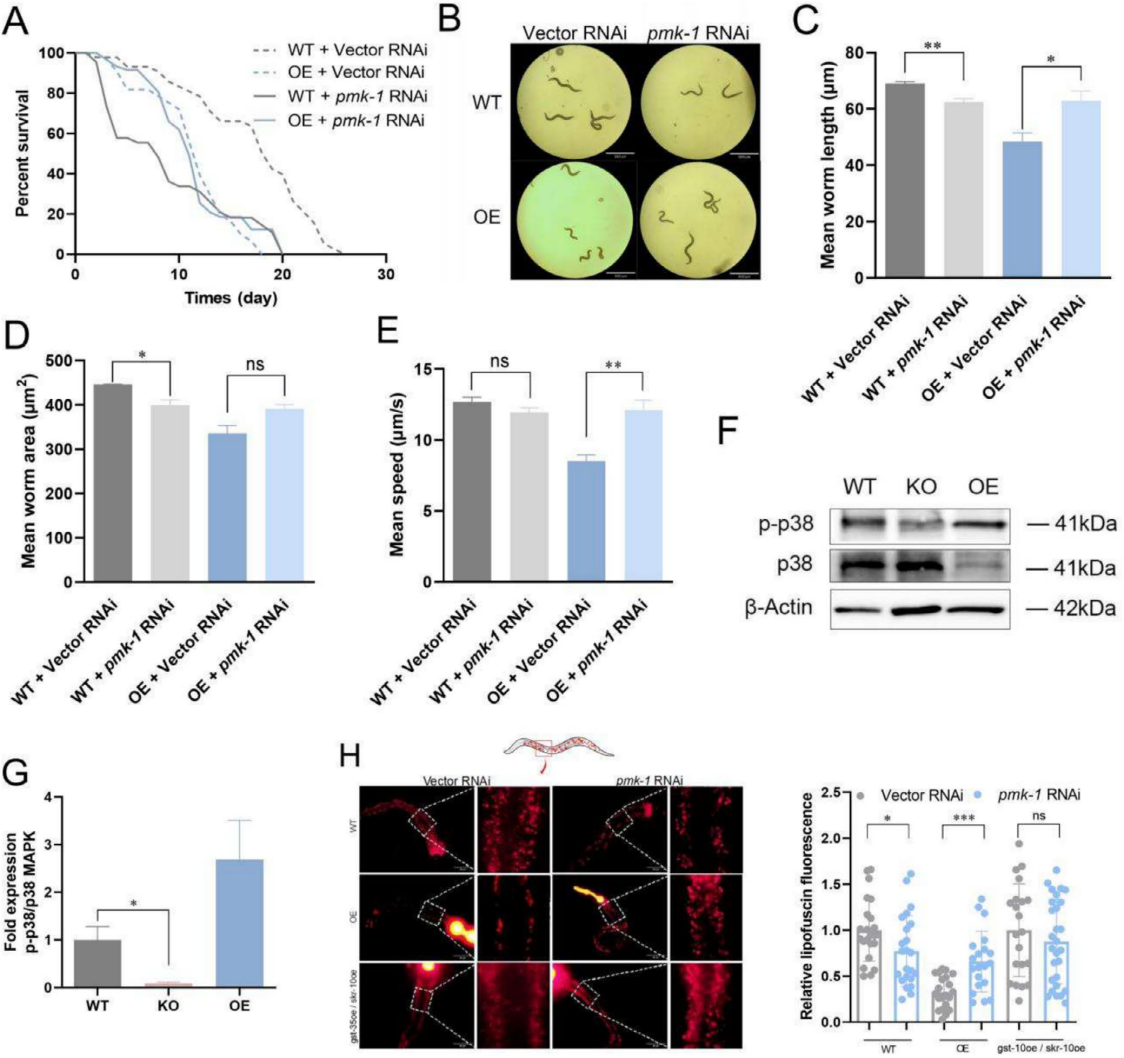
*Gst‐35*oe impacts the health span of nematodes by stimulating the PMK‐1 pathway. (A) The survival curves of WT and OE nematodes fed either vector L4440 control bacteria or bacteria expressing RNAi for *pmk‐1*. (B–E) The body length, body area, and moving speed of WT and OE nematodes fed vector L4440 control bacteria or bacteria expressing RNAi for *pmk‐1* on Day 1. (F, G) Western blotting of p‐p38 mitogen–activated protein kinase (MAPK) protein expression levels in WT, KO, and OE nematodes. (H) Representative fluorescence images of WT, OE, and *gst‐35*oe/*skr‐1*0oe nematodes (treated with vector RNAi or *pmk‐1* RNAi) stained with LysoTracker Red on Day 1 and its quantitative determination of acidic fluorescence intensity in the intestine. Data were represented as mean ± SEM of three independent experiments. *p < 0.05; **p < 0.01; ***p < 0.001 vs. OE with vector RNAi.

The inflammatory process involves activation of the p38 mitogen–activated protein kinase (MAPK) signaling pathway, which induces oxidative stress and triggers an inflammatory cascade that accelerates aging (Anerillas et al. 2020; Wang et al. 2024). Inhibition of p38 MAPK exhibits anti‐inflammatory effects and has therapeutic potential for inflammation‐associated diseases, thereby delaying aging (Wang et al. 2024; Yeung To et al. 2018). In this study, the lifespan of OE nematodes treated with *pmk‐1* RNAi showed no further reduction compared to controls, whereas the lifespan of WT nematodes treated with *pmk‐1* RNAi was significantly reduced (Figure 5A and Table 2). These findings suggested that *gst‐35oe* induces activation of the PMK‐1/p38 pathway, contributing to the reduced lifespan in OE nematodes. Thus, RNAi targeting *pmk‐1* did not exacerbate this reduction. Conversely, as *pmk‐1* is essential for survival (Zarubin and Han 2005), its suppression in WT nematodes resulted in a dramatic lifespan decrease. The inability to extend lifespan through *pmk‐1* RNAi further implied that the detrimental effects of *gst‐35*oe might partially arise from PMK‐1/p38 activation. This hypothesis was supported by a significant restoration of the body length, body area, and movement speed in OE nematodes following *pmk‐1* RNAi treatment (Figure 5B–E). Additionally, phosphorylation of the PMK‐1/p38 protein was markedly elevated in OE nematodes compared to WT nematodes (Figure 5F,G). Since inflammation, lipofuscin accumulation, and aging are known to damage lysosomes (Park et al. 2018; Tirpude et al. 2022), inhibiting p38 may restore lysosomal function, enhance autophagy, and slow aging (Park et al. 2018, 2022). Consistent with this, LysoTracker Red staining revealed that lysosomal activity in *pmk‐1* RNAi OE nematodes significantly improved on Day 1 (Figure 5H), indicating that lysosomal dysfunction in OE nematodes depends on PMK‐1/p38 activation. However, silencing *pmk‐1* did not enhance lysosomal activity or physiological metrics in *gst‐35*oe/*skr‐10*oe nematodes (Figure 5H and Figure S2). This suggests that *gst‐35*oe compromises the health span of nematodes by activating PMK‐1/p38 and simultaneously downregulating *skr* genes.

**TABLE 2:** Lifespan of WT and OE nematodes fed vector L4440 control bacteria or bacteria expressing RNAi for *pmk‐1* at 20°C.

Strain	Number of nematodes	Mean lifespan (day)	Change in lifespan (%)
WT + vector RNAi	108	15.87 ± 0.72	
OE + vector RNAi	96	11.09 ± 0.28**	−30.12
WT + *pmk‐1* RNAi	115	7.78 ± 0.31***	−50.98
OE + *pmk‐1* RNAi	109	10.59 ± 0.12**	−33.27

*Note:* The statistical difference between the curves was analyzed using the log‐rank test.

** *p* < 0.01.

*** *p* < 0.001 vs. WT + vector RNAi.

## 3 Discussion

Recognizing the importance of maintaining health while addressing aging is paramount (Guan and Yan 2022). Aging is typically accompanied by metabolic abnormalities, chronic inflammation, protein homeostasis disruption, lysosomal dysfunction, and other hallmarks that contribute to age‐related diseases (Campisi et al. 2019; Levine and Kroemer 2019; López‐Otín et al. 2023). Decades of extensive research have identified numerous genetic targets for extending lifespan. However, these advances often fail to improve health span concurrently (Rollins et al. 2017). Therefore, achieving the dual goals of prolonged lifespan and extended health span remains a critical challenge. Our study demonstrated that *gst‐35*oe induced lysosomal dysfunction and reduced activity by upregulating the *pmk‐1* signaling pathway and downregulating *skr* genes. This led to significant lifespan reduction, physiological impairments, metabolic disruptions, and other health complications. Conversely, although the *gst‐35* mutant did not extend lifespan, certain health indicators showed partial improvement. These findings suggest that *gst‐35* plays a pivotal role in regulating the health span of *C. elegans* .

The GST gene superfamily consists of seven classes: alpha, mu, pi, sigma, theta, omega, and zeta, with the pi (GSTP), mu (GSTM), and theta (GSTT) classes being the most extensively studied (van de Wetering et al. 2021). In contrast, the role of the alpha class (GSTA) in promoting healthy longevity remains unexplored. Historically, GST enzymes have been associated with detoxifying electrophiles through glutathione conjugation (Strange et al. 2001). However, growing evidence suggests that OE of GST enzymes may contribute to the onset of various disorders, including cancer, Alzheimer's disease (AD), Parkinson's disease (PD), asthma, and epilepsy (Dai et al. 2021; Lv et al. 2023). One explanation is the dual role of GST enzymes, which exhibit both anti‐inflammatory and pro‐inflammatory effects. For instance, GST enzymes can exacerbate PD and AD by enhancing interleukin‐1β activity (Li et al. 2003) and worsen asthma progression through PGD2 production (Dai et al. 2021). Similarly, GSTA1‐3 expression levels are significantly elevated in age‐related diseases such as diabetes, atherosclerosis, and AD (Lin et al. 2017; Niu et al. 2021; Rooney et al. 2023; Wang et al. 2023). To date, research on GST homologs in *C. elegans* has primarily focused on *gst‐4* and *gst‐14* (Hu et al. 2021; Suthammarak et al. 2013). Previous studies have shown that GSTA1‐3 expression increases with age in mice (Patel et al. 1998), particularly in cancer models, suggesting that GSTA1‐3 may negatively impact health during aging. Consistent with these findings, our study revealed that the expression of *gst‐35*, the *C. elegans* homolog of GSTA1‐3, significantly increases with age (Figure 1). These results imply that *gst‐35* may detrimentally affect health as nematodes age.

Aging is characterized by chronic activation of immune responses, as evidenced by a high circulating level of inflammatory markers, a condition called “inflammaging,” which is associated with an increased risk of age‐related pathologies and disabilities (Singh et al. 2024). Excessive GST expression can deplete glutathione reserves (Rooney et al. 2023), reducing cellular ROS scavenging capacity and triggering oxidative stress (Hellou et al. 2012). Chronic oxidative stress can further induce a persistent inflammatory state, driving the progression of age‐related diseases such as cancer, diabetes, and inflammatory disorders (Borgoni et al. 2021; Izvolskaia et al. 2020; Mittal et al. 2014; Ward et al. 2023). Notably, activation of GSTA enzymes has been shown to stimulate ROS production (Garige and Walters 2015). Our study revealed that *gst‐35*oe reduced GSH/GSSG ratio, impaired antioxidant capacity, led to excessive ROS accumulation, and promoted the production of the inflammatory mediator PGD2 (Figure 3). These findings align with prior studies indicating that *gst‐14*, a paralog of *gst‐35*, plays a role in the nematode inflammatory response (Suthammarak et al. 2013). Moreover, ROS is a key factor in maintaining physiological homeostasis (Davalli et al. 2016); excessive ROS can trigger lipid peroxidation cascades, resulting in impaired oocyte quality and adversely affecting reproductive and developmental functions (Yong et al. 2021). Consistent with this, we observed impaired reproductive development in *gst‐35*oe nematodes (Figure 2). Collectively, these findings indicate that *gst‐35*oe drives ROS accumulation, reduces stress resistance, induces an inflammatory state, and compromises both lifespan and health span in nematodes.

Lysosomes are pivotal organelles for cellular catabolism, acting as degradation and signaling hubs essential for maintaining homeostasis, development, and aging (Li et al. 2024; Mutvei et al. 2023). Oxidative stress is a key contributor to autophagy and lysosomal dysfunction, accelerating aging and impairing health (Filomeni et al. 2015; Levine and Kroemer 2019). Elevated ROS levels disrupt lysosomal acidification, leading to enzymatic inactivation, impaired degradation capacity, lysosomal membrane instability, and structural damage (He et al. 2023). Evidence from both invertebrate and mammalian models underscores the presence of lysosomal dysfunction in aging (Kaushik et al. 2021). Lysosomal dysfunction impairs the fusion of autophagosomes with lysosomes, leading to the accumulation of undigested materials, including lipofuscin—an established marker of aging (Stoka et al. 2016). Additionally, lysosomal protein hydrolysis disruptions can compromise protein homeostasis, a hallmark of aging (Stoka et al. 2016). Lysosomal activation also plays a critical role in regulating reproductive and developmental processes (Zeng et al. 2023; Zou et al. 2024). Although the link between GST and lysosomal regulation remains underexplored, prior research has implicated GST in autophagy regulation (Lv et al. 2023). Our transcriptomic analysis revealed significant enrichment of lysosomal gene expression in *gst‐35*oe nematodes (Figure 4E,F). Functional assessments indicated lysosomal dysfunction in OE nematodes (Figure 4K). OE of lysosomal genes such as *mig‐23*/ENDIP7, *cpr‐3*/cathepsin B, *asp‐3*/cathepsin D, and *heh‐1*/NPC2 has been reported to trigger aging effector factors, reduce lysosomal activity, enhance lipid accumulation, and accelerate aging (Choi et al. 2024; Hook et al. 2022; Stoka et al. 2016; Wen et al. 2019). These results suggest that *gst‐35*oe accelerates aging and disrupts healthy physiological parameters in nematodes by impairing lysosomal function.

The downregulated genes in our study were significantly enriched in pathways such as TGF‐β, Wnt, and ubiquitin‐mediated protein degradation (Figure 4F). Among these, most downregulated genes belong to the *skr* gene family, homologous to SKP1 in mammals. SKP1 phosphorylation has been shown to positively regulate autophagy, playing a key role in protein ubiquitination and degradation (Li, Krause, et al. 2023). OE of SKP1, however, can disrupt normal cellular physiology, suggesting that SKP1 expression must remain within a specific threshold for optimal functionality (Thompson et al. 2022). SKP1 has a dual role, acting as both a protective and a detrimental factor depending on cellular conditions. Our findings revealed that *skr* gene expression was significantly reduced in both KO and OE nematodes compared to WT nematodes (Figure 4J). Specifically, lysosomal activity was enhanced in KO nematodes but reduced in OE nematodes (Figure 4K). Quantitative analysis showed that the average mRNA levels of *skr* genes were reduced by approximately 49.62% in KO nematodes and by 76.69% in OE nematodes (Table S4). These results suggest that the degree of *skr* genes downregulation may underlie the differential lysosomal activity observed in KO and OE nematodes. Previous studies have shown that SKP1 KO is lethal in mice (Zhou et al. 2013), whereas silencing *skr* genes in *C. elegans* delays growth and development (Nayak et al. 2002; Yamanaka et al. 2002), a phenotype also observed in our study (Figure S1). Partial reduction (~50%) of SKP1 expression alleviates inflammation (Li, Sano, et al. 2023). Together, these findings suggest that *skr*/SKP1 operates within a critical threshold for effective biological function. Interestingly, upregulating *skr‐10* expression in OE nematodes partially restored their health span (Figure 4M,N), highlighting that *gst‐35*oe significantly suppresses *skr* genes expression, disrupting lysosomal function and negatively impacting nematode lifespan.

RNAi targeting *pmk‐1* significantly shortened the lifespan of WT nematodes, whereas OE nematodes exhibited only slight delays in lifespan reduction (Figure 5A). The p38 MAPK signaling pathway, activated by pro‐inflammatory and stress stimuli, plays a conserved role in innate immunity in nematodes (Kim et al. 2022; Yong et al. 2009). Lysosomal dysfunction caused by inflammation, lipofuscin accumulation, and aging can be alleviated by inhibiting p38, which restores lysosomal activity, promotes autophagy, and slows aging (Anerillas et al. 2020; Wang et al. 2024; Yeung To et al. 2018). Additionally, p38 mediates cellular senescence in response to telomere shortening and oxidative stress (Zarubin and Han 2005). However, due to its essential role in cellular development and the cell cycle, p38 KO is lethal in mice (Zarubin and Han 2005). Our results showed elevated PMK‐1/p38 phosphorylation in OE nematodes, whereas *pmk‐1* silencing restored development, motility, and lysosomal activity in these worms (Figure 5). These findings suggest that *gst‐35*oe induces inflammation by hyperactivating the p38 MAPK pathway, leading to compromised physiological states in nematodes, and that reducing PMK‐1/p38 activity can reverse *gst‐35*oe‐induced damage. This may explain why *pmk‐1* silencing failed to further shorten the lifespan of OE nematodes. In contrast, WT nematodes treated with *pmk‐1* RNAi exhibited growth and developmental defects, resulting in significantly shortened lifespans. Additionally, SKP1 homologs *skr‐1/2* are essential for *C. elegans* SKN‐1‐mediated antioxidant and detoxification responses, functioning independently of the p38 MAPK pathway (Wu et al. 2016). Consistent with this, our results showed no significant alterations in lysosomal activity or physiological markers when *skr‐10*oe was combined with *pmk‐1* suppression in *gst‐35*oe nematodes. These findings indicate that *gst‐35* mediates lysosomal dysfunction through two distinct pathways: the *pmk‐1* pathway and the *skr* genes. Together, these pathways contribute to metabolic inefficiency, immune overload, stunted growth, reduced reproduction, and health span decline in nematodes.

This study investigates the impact of *gst‐35*/GSTA1‐3 on nematode health span, offering new insights into its mechanisms and potential therapeutic implications. Unlike previous research focusing on GSTA1‐3 in disease contexts, our work highlights its role in aging‐related physiological changes. Although *C. elegans* offers a valuable model for studying aging, its relevance to other organisms, including mammals, remains limited. Future studies should expand upon these findings by utilizing diverse model organisms and larger sample sizes. Translating these findings to human health will require more relevant models or human‐centric studies. Our ongoing research aims to address these limitations by investigating downstream targets and enzymes linked to aging. By overcoming these challenges, we hope to deepen our understanding of GST proteins in aging and develop novel therapeutic strategies to promote healthy aging.

## 4 Experimental Procedures

### 4.1 Strains and Maintenance

*Caenorhabditis elegans* strains were maintained at 20°C on nematode growth medium (NGM) plates seeded with the *Escherichia coli* OP50 feeding strain. WT (N2) nematodes, provided by Caenorhabditis Genetics Center (University of Minnesota, USA), were used in this study. The PHX6971 *gst‐35* (syb6971) was generated by SunyBiotech (Fujian, China) and features a 1583 deletion of the Y1H11.2.1 region, encompassing the ATG to the TAA stop codon. This strain was outcrossed four times. Additionally, strains PHX7528 sybIs7528 (Pdpy‐30::GFP::T2A::*gst‐35*::unc‐54 3′UTR, Pmyo‐2::mCherry) and HBW007 (Pdpy‐30::GFP::T2A::*gst‐35*::unc‐54 3′UTR, Pdpy‐30::GFP::T2A::*skr‐10*::unc‐54 3′UTR, Pmyo‐2::mCherry) were generated by SunyBiotech.

### 4.2 RNAi Constructs for *C. elegans*

RNA interference (RNAi) experiments were based on a previously reported protocol (Li et al. 2018). The hermaphrodite nematodes were fed *E. coli* (HT115) containing either an empty control vector (L4440) or expressing double‐stranded RNAi. Genomic fragments were polymerase chain reaction (PCR) amplified to cover the second exon of *pmk‐1, skr‐7, skr‐8*, and *skr‐10* to generate the *pmk‐1, skr‐7, skr‐8*, and *skr‐10* RNAi construct. Subsequently, the resulting amplicon was cloned into the plasmid L4440. RNAi experiments were performed using tetracycline‐resistant *E. coli* (HT115) carrying double‐stranded RNA against *pmk‐1, skr‐7, skr‐8, skr‐10*, or empty vector control (pL4440). The *E. coli* HT115 (DE3) bacterial colony containing either pL4440 or the plasmid with the target gene was inoculated in a Luria Bertani (Li et al.) broth containing 100 μg/mL ampicillin (Sigma, Shanghai, China) and 12.5 μg/mL tetracyclines and grown for 8 h in a shaker at 37°C. All RNAi constructs were sequence verified using the primer 5′‐TGTAAAACGACGGCCAGT. Synchronized populations of L1‐stage nematodes were allowed to develop at 20°C on seeded NGM agar plates.

### 4.3 Lifespan Assay

Two techniques for life testing were used in this study, namely, the liquid and solid culture life experiments. A liquid culture life experiment was used to assess the effect of *gst‐35* on nematode longevity. Synchronized populations of worms were transferred into 96‐well plates (10–12 per plate) together with the S‐complete medium, *E. coli* OP50, and 50 μg/mL carbenicillin. Subsequently, 200 μM 5‐fluoro‐2′‐deoxyuridine (FUDR) (Sigma, Shanghai, China) was added after 48 h. Thus, progeny development was blocked at the L4 stage. The L4 stage was considered on Day 0 of their lifespan. Next, worms were observed and scored every 2 days for survival. The differences in the developmental stages of nematodes were observed and recorded. *E. coli* OP50 was added during assays to avoid starvation.

To study the lifespan of RNAi‐treated nematodes using a solid culture life experiment method, we initially inoculated cultured *E. coli* containing RNAi strains onto 3‐cm NGM agar plates with 1 mM isopropyl β‐D‐thiogalactoside (Sigma, Shanghai, China). Subsequently, the synchronized population nematodes induced by RNAi strains were cultured on agar plates. After 48 h of cultivation, the nematodes were transferred to *E. coli* RNAi strain plates containing 33 mM FUDR to prevent hatching. The L4 stage was designated as Day 0 of their lifespan. Nematodes that did not respond to gentle mechanical stimuli were scored as dead. Nematodes were transferred into a new NGM agar plate containing RNAi strains every 2 days to prevent starvation.

### 4.4 Measurement of Body Length, Body Area, and Movement Assay of Nematodes

Synchronized nematodes were transferred into 96‐well plates (10–12 per plate) together with the S‐complete medium, *E. coli* OP50, and 50 μg/mL carbenicillin. FUDR (200 μM) was added to block progeny development at the L4 stage. Owing to the slower development of PHX7528, we designated the initial day of the adult phase as Day 1, and this protocol was consistently applied in subsequent trials without further modification. Adult worms on Days 1 and 5 were transferred to NGM plates without food, and the body length, body area, and mean speed per worm in 10 s were recorded by WormLab (MBF Bioscience, Williston, VT, USA). This assay was repeated independently thrice, and each group included at least 30 worms.

### 4.5 Fertility Assay

The fertility experiment involved recording the initial fertility time, the number of eggs laid, and the incubation rate of eggs. First, the synchronized nematodes were cultivated until they reached the L3 stage and transferred to fresh 30 mm NGM plates seeded with OP50. Each plate contained one worm, and each nematode type had eight replicates. After labelling, the nematodes were cultivated at 20°C. The spawning status of the worms was monitored every hour until egg laying stopped. The exact fertility time was recorded as the initial spawning time. Subsequently, the number of eggs on the initial plate was counted at 24‐h intervals until egg production stopped. Simultaneously, the nematodes were transferred into the freshly seeded NGM plates with OP50. After 48 h, the hatching of nematode eggs was observed and recorded.

### 4.6 Body Bend Assay

To measure the body bending frequency, synchronized L4 larvae were treated as described in the body length assay. To inhibit the growth of offspring at the L4 stage, FUDR (200 μM) was added. Adult worms were transferred into 96‐well plates (one per plate) without food and scored for the number of body bends in 30 s. Each treatment included 30 nematodes, and this assay was performed thrice independently.

### 4.7 Measurement of Age and Pigment Level

The nematodes were positioned on slides and rendered unconscious using 20 mM levamisole. The fluorescent microscope (Olympus BX53, Japan) was used to view and capture images of the spontaneous fluorescence in the intestines of worms. The fluorescence intensities of the age pigment were quantified using the ImageJ 1.53t software and normalized to the body area of the worms.

### 4.8 Food Intake Assay

Food intake was assessed under liquid culture. Optically clear 96‐well plates (Costar, USA) were prepared, each containing a total volume of 100 μL of S‐complete medium, *E. coli* OP50, and 10 drug‐treated worms. Each plate was placed on a plate shaker for 1–2 min before counting. The optical density at 600 nm (OD600) of each well was determined (Gomez‐Amaro et al. 2015). Measurements were taken on Days 0 and 5. This assay was independently repeated thrice, and each treatment included 30 nematodes.

### 4.9 Oxidative Stress Assay

For the oxidative stress assay, synchronized N2, PHX6971, and PHX7528 worms were pretreated as described in the body length assay. To inhibit the development of offspring at the L4 stage, FUDR (200 μM) was added. Worms were transferred into a new 96‐well plate containing 50 mM paraquat on Day 5. The nematode's survival was monitored daily. Worms that exhibited no response to mechanical stimuli were presumed to be dead. Each assay was independently conducted in three separate trials. Each group comprised a minimum of 30 worms.

### 4.10 Measurement of Intracellular ROS

The ROS levels within the cells were quantified using the fluorescent probe 2′,7′‐dichlorodihydrofluorescein diacetate (H_2_DCF‐DA) (Beyotime Biotechnology, Shanghai, China) (Yoon et al. 2018). The worms were subjected to a body length assay. On Day 5 of their adult stage, nematodes were rinsed thrice with M9 buffer to eliminate the remaining bacteria. Subsequently, they were transferred to black 96‐well plates, which contained 500 μM H_2_DCF‐DA. The nematodes were incubated at 37°C for 2 h. The fluorescence was subsequently quantified using excitation and emission wavelengths of 485 and 530 nm, respectively. The experiment was performed in triplicates with 30 worms used for each replicate.

### 4.11 *P. aeruginosa* (PA14) Maintenance and Infection Assays

We used the pathogenic bacterium *P. aeruginosa* PA14 for nematode treatment following the big‐lawn method of Park et al. (2017). PA14 was cultured in LB at 37°C overnight. Colonies of PA14 were picked and cultured for approximately 16 h. Subsequently, 20 μL of activated PA14 was uniformly spread on a 35‐mm NGM plate using a coating rod. The NGM plates were inverted and incubated at 37°C for 24 h to allow PA14 to exhibit robust virulence. Thirty newly synchronized populations of nematodes were transferred to NGM plates coated with PA14, and the process was repeated thrice. Nematodes were observed every 24 h, and their responsiveness was assessed by gently touching their heads with a platinum wire. Nematodes that displayed no movement were considered dead. Prompt observations were crucial, as PA14 can dissolve the nematode, whereas the presence of fungal moss on the plates required careful attention to ensure accurate nematode detection.

### 4.12 Measurement of PGD2

Populations of N2, PHX6971, and PHX7528 strains were synchronized and cultivated on NGM plates at 20°C until they reached Day 5 of the adult stage. Subsequently, the nematodes were harvested in phosphate‐buffered saline (PBS). PGD2 was detected using enzyme‐linked immunosorbent assay kits (Upingbio, Shenzhen, China). The experiment was repeated thrice, with 2000 worms used in each replication.

### 4.13 Determination of Reduced and Oxidized Glutathione (GSH/GSSG) Ratio

The GSH/GSSG ratio is a significant indicator of the redox environment and its associated cellular health (Ferguson and Bridge 2019). At least 1000 worms each was collected on Days 1 and 5. They were washed thrice with M9. The harvested nematodes were frozen at −80°C for at least 2 h. The GSH/GSSG ratio was determined using the Reduced and Oxidized GSH Content Kits (Upingbio, Shenzhen, China). The experiment was repeated thrice.

### 4.14 RNA Sequencing

The growth of N2, PHX6971, and PHX7528 strains was synchronized, and their eggs were allowed to hatch and develop to the young adult stage on NGM plates at 20°C. On Day 5 of the adult stage, the nematodes were harvested in PBS. Gene expression was assessed by Novo Gene Corporation (Beijing, China). Sequencing libraries were generated using NEBNext Ultra RNA Library Prep Kit for Illumina (New England Biolabs, Ipswich, MA, USA) following the manufacturer's recommendations, and index codes were added to attribute sequences of each sample. To select cDNA fragments of 250–300 bp length, the library fragments were purified using the AMPure XP system (Beckman Coulter, Beverly, USA). Subsequently, 3‐μL USER Enzyme (New England Biolabs) was used with the size‐selected, adaptor‐ligated cDNA at 37°C for 15 min, followed by 5 min at 95°C before PCR. Next, PCR was performed with phusion high‐fidelity DNA polymerase, universal PCR primers, and Index (X) Primer. Finally, PCR products were purified (AMPure XP system), and library quality was assessed on the Agilent Bioanalyser 2100 system. The clustering of the index‐coded samples was performed on a cBot Cluster Generation System using the TruSeq PE Cluster Kit v3‐cBot‐HS (Illumina) according to the manufacturer's instructions. After cluster generation, the library preparations were sequenced on an Illumina NovaSeq platform, generating 150 bp paired‐end reads.

Up‐ or downregulated genes were identified by filtering RNA‐seq data with a cutoff of a two‐fold change in expression level and a false discovery rate analog of *p* < 0.05. The Kyoto Encyclopaedia of Genes and Genome (KEGG) pathway enrichment was performed using “clusterProfiler” with a significance criteria of *p* < 0.05 (Yu et al. 2012). All raw data and detailed experimental methods have been uploaded to GEO database, GEO number: GSE273339.

### 4.15 RNA Isolation and qPCR

The worms were initially subjected to the body length assay. *E. coli* OP50 was washed off with M9, and the worms were frozen at −80°C for at least 2 h. Total RNA was isolated using Trizol (Tiangen, China), and cDNA was synthesized using a cDNA synthesis kit (TaKaRa Bio, Dalian, China). qPCR was conducted using SYBR Premix Ex Taq (TaKaRa Bio, Kyoto, Japan) on a LightCycler96 Real‐time PCR system (Roche, Switzerland). Subsequently, actin‐1 expression was used as an endogenous control to normalize the mRNA quantity obtained from a target gene. Samples were run in triplicate, and the primers are listed in Table S1.

### 4.16 LysoTracker Staining

*Caenorhabditis elegans* were grown from hatch to Day 1 of adulthood on OP50‐bacteria–seeded plates containing 25 μM LysoTracker DeepRed (Beyotime Biotechnology, Shanghai, China) (Baxi and de Carvalho 2018). Subsequently, worms were imaged with an upright fluorescence microscope (Olympus) using 8–10 animals per genotype and three independent biological replicates. Quantification was performed using ImageJ 1.53t, and statistical analysis was conducted using a one‐way analysis of variance with the Tukey's multiple comparisons test.

### 4.17 Western Blotting

The N2, PHX6971, and PHX7528 strains were treated as in the RNA‐seq assay, ensuring that the total count of nematodes was ≥ 2000. The nematodes were washed with M9 buffer to remove the *E. coli* OP50 and drugs and collected in 1× PBS at −80°C for 2 h to facilitate cracking. Total protein was extracted with ice‐cold radioimmunoprecipitation assay lysis buffer (Beyotime Biotechnology, China) containing 1× protease inhibitor and 1x phosphatase inhibitor cocktail (Beyotime Biotechnology), and loading buffer was added after protein quantification using the bicinchoninic acid kit (Thermo Fisher Scientific, America) to prevent protein degradation. A 10–180 kDa protein maker (Solarbio, China) was used as an indicator of molecular weight. Protein samples were run at 30 V for 40 min on a stacking gel and at 80 V for 120 min on a separating gel. p‐p38 MAPK (Thr180/Tyr182) polyclonal antibody (dilution 1:1000, Proteintech) was used to determine the p‐p38 levels. For normalization, membranes were stripped with stripping buffer (Servicebio, China) and incubated with p38 MAPK polyclonal antibody (dilution 1:1000, Proteintech); they were restripped and incubated with β‐actin monoclonal antibody (dilution 1:1000, Proteintech). Protein bands were detected using the standard enhanced chemiluminescence western blotting substrate. The mean densities of the bands were analyzed using ImageJ 1.53t.

### 4.18 Statistical Analysis

GraphPad Prism 8 software and ImageJ 1.53t were used for statistical analysis. Lifespan and stress resistance were analyzed using Kaplan–Meier survival methods. Log‐rank tests were used to determine significant differences. For other measurements, *p*‐values were tested using the Student's *t*‐test. *p* < 0.05 was considered a significant difference.

## Author Contributions

Yehui Gao: conceptualization, methodology, data curation, formal analysis, investigation, writing – original draft. Xinyun Zhang, Congmin Wei, and Hongru Lin: methodology, validation. Mengchen Wu, Botian Ma, and Jinyun Jiang: data curation, formal analysis. Shan Li: project administration, supervision, resources, writing – review and editing. Hongbing Wang: project administration, conceptualization, funding acquisition, supervision, resources, writing – review and editing. All authors have approved the final article.

## Conflicts of Interest

The authors declare no conflicts of interest.

## Supporting information


Appendix S1

## Data Availability

Data will be made available on request.
