# Supplementary material for: Negative Effect of Gst‐35 on the Health Span of Caenorhabditis elegans Through Lysosomal Dysfunction via the Pmk‐1 and Skr Genes
Source: Aging Cell. 2025 Feb 13;24(6):e70016. doi: 10.1111/acel.70016 (PMC12151881; doi:10.1111/acel.70016)
Supplement: Supplementary file 1 — Appendix S1 [file ACEL-24-e70016-s001.docx]

***Negative effect of gst-35 on the health span of Caenorhabditis elegans through lysosomal dysfunction via the pmk-1 and skr genes***

**Table S1.** List of primers used for the quantitative real-time reverse transcription-polymerase chain reaction.

**Table S2.** The average fist egg-laying time, number of eggs laid and hatching rate of WT, KO and OE nematodes at 20°C*.*

**Table S3.** Downregulated differential genes enriched in TGF-β signaling pathway, Wnt signaling pathway and ubiquitin mediated proteolysis.

**Table S4.** Percentage of *skr* genes downregulation in KO and OE nematodes

**Figure S1.** Silencing *skr-7, skr-8,* and *skr-10* genes has been shown to impair the growth and development levels of nematodes.

**Figure S2.** Silencing *pmk-1* expression did not influence the growth and developmental capacity of *gst-35*oe/*skr-10*oe nematodes.

**Table S1.** List of primers used for the quantitative real-time reverse transcription-polymerase chain reaction

| Gene name | Primer sequence |
| --- | --- |
| *gst-35* | Forward: CCTACTTCGACATCCGAGCG |
|  | Reverse: AGGTAGCGTTGGATAGCAGC |
| *mig-23* | Forward: GCAGGGAAGACGACTCGTTA |
|  | Reverse: AGTTTCCGGTACCTCTTCGCA |
| *cpr-3* | Forward: TGGAGGTCACGCGGTTAAAA |
|  | Reverse: TACGTTTCCGAGTGCGTTCC |
| *vha-15* | Forward: GGCGGAAGTTCCTCATCACA |
|  | Reverse: CGCGGGATTTGTCTTCCTGA |
| *asp-3* | Forward: ACAACGATGTTGTCTGCTTCG |
|  | Reverse: TTTCCGACGTAGTGGTTCGG |
| *pho-11* | Forward: CTCCCCACTCACTTGGTTCC |
|  | Reverse: GTTCTGACTAGCGGCACACT |
| *heh-1* | Forward: TCGGATGCTTGTACTTATGGTGT |
|  | Reverse: GGGACGAGTAAGCTGCCAAT |
| *W07B8.4* | Forward: TAACTGCGGCGATGGATGTG |
|  | Reverse: TCCGGTGACTAGTCCGTTCT |
| *pho-4* | Forward: TGATGGTTCAGGCGATTTGGA |
|  | Reverse:  AATTGTCCCCAACCACCTCC |
| *skr-8* | Forward: TCGTTGCCTGCAACTACCTT |
|  | Reverse: GTTGATGGTTCAGCTGCGTC |
| *skr-7* | Forward: TCCAATTGTCGCCCCAATCA |
|  | Reverse: AAGTCGAAGAGCACGTCGTT |
| *skr-10* | Forward: CCAAGAAGCTCCAGTTGCTG |
|  | Reverse: TGCAGGCAACAATCAAGTCG |
| *skr-15* | Forward: GCGAGCAAGCCATCAAACAA |
|  | Reverse: ATTCCTTTTGCCAAACCCGC |
| *skr-13* | Forward: GAGAAGCACAAAGCCGATGC |
|  | Reverse: AGCAGCATCCTGTTCATCCG |
| *skr-9* | Forward: CGGTCGAAGTTCAAGCCAAC |
|  | Reverse: AGAGCACGTCGTTGTCGATT |
| *skr-14* | Forward: TCCAGTTTCCGATGCTCCAG |
|  | Reverse: CTCACACCATTCAGCGACCT |
| *actin-1* | Forward: TCGGTATGGGACAGAAGGAC |
|  | Reverse: CATCCCAGTTGGTGACGATA |

**Table S2.**

The average fist egg-laying time, number of eggs laid and hatching rate of WT, KO and OE nematodes at 20°C

| Strain | Average fist egg-laying time (h) | Average number of eggs laid (pc) | Average hatching rate (%) |
| --- | --- | --- | --- |
| WT | 59.00 ± 0.71 | 342.40 ± 20.19 | 98.58 ± 0.25 |
| KO | 59.75 ± 1.00 | 315.60 ± 12.36 | 98.52 ± 0.24 |
| OE | 88.00 ± 2.78**** | 136.30 ± 12.02**** | 65.60 ± 3.86**** |

Date represent mean ± SEM of three independent experiments. ****P<0.0001 vs WT.

**Table S3.** Downregulated differential genes enriched in TGF-β signaling pathway, Wnt signaling pathway and ubiquitin mediated proteolysis.

| TGF-β signaling pathway | Wnt signaling pathway | Ubiquitin mediated proteolysis |
| --- | --- | --- |
| *skr-13* | *skr-13* | *skr-13* |
| *skr-9* | *skr-9* | *skr-9* |
| *skr-10* | *skr-10* | *skr-10* |
| *skr-15* | *skr-15* | *skr-15* |
| *skr-14* | *skr-14* | *skr-14* |
| *skr-8* | *skr-8* | *skr-8* |
| *skr-7* | *skr-7* | *skr-7* |
|  | *mom-1* |  |

**Table S4.** Percentage of *skr* genes downregulation in KO and OE nematodes

| Gene ID | Strains | |
| --- | --- | --- |
|  | KO | OE |
| *skr-13* | 49.39% | 76.89% |
| *skr-9* | 16.80% | 89.67% |
| *skr-10* | 56.82% | 68.91% |
| *skr-15* | 53.75% | 78.11% |
| *skr-14* | 63.08% | 84.31% |
| *skr-8* | 49.18% | 68.35% |
| *skr-7* | 58.36% | 70.57% |
| Average value | 49.62% | 76.69% |


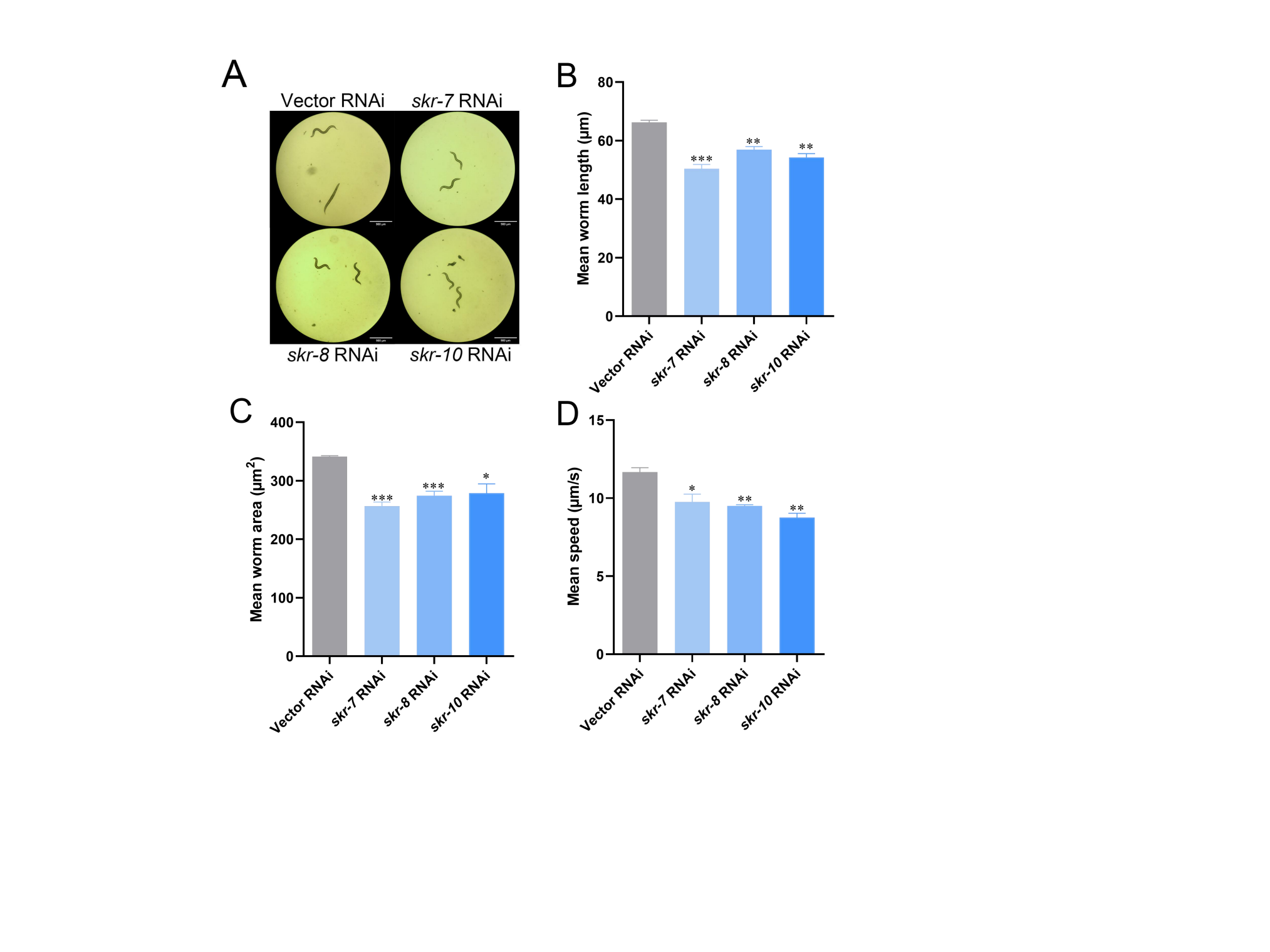


**Figure S1.** Silencing *skr-7, skr-8,* and *skr-10* genes has been shown to impair the growth and development levels of nematodes. (A-D) The body length, body area, and moving speed of WT nematode fed vector L4440 control bacteria or bacteria expressing RNAi for *skr-7, skr-8,* and *skr-10* on day 1. *P<0.05; **P<0.01; ***P<0.001 vs WT with vector RNAi.


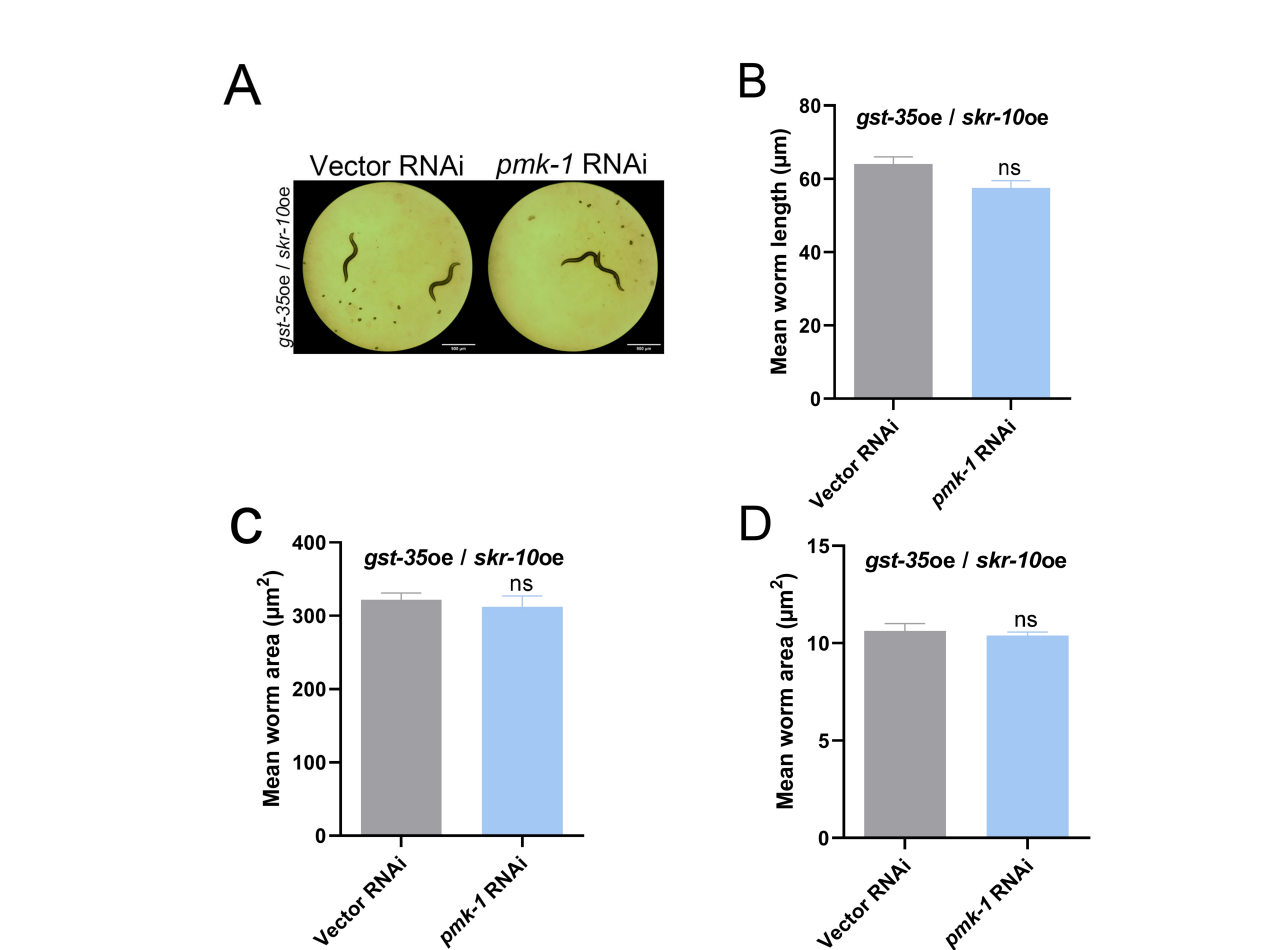


**Figure S2.** Silencing *pmk-1* expression did not influence the growth and developmental capacity of *gst-35*oe/*skr-10*oe nematodes. (A-D) The body length, body area, and moving speed of *gst-35*oe/*skr-10*oe nematode fed vector L4440 control bacteria or bacteria expressing RNAi for *pmk-1* on day 1.
